# Adipokines and Sexual Hormones Associated with the Components of the Metabolic Syndrome in Pharmacologically Untreated Subjects: Data from the Brisighella Heart Study

**DOI:** 10.1155/2011/724816

**Published:** 2011-11-09

**Authors:** Arrigo F. G. Cicero, Paolo Magni, Massimo Moré, Massimiliano Ruscica, Elena Dozio, Liliana Steffani, Claudio Borghi, Felice Strollo

**Affiliations:** ^1^Department of Internal Medicine, Aging and Kidney Disease Department, University of Bologna, 40138 Bologna, Italy; ^2^Department of Internal Medicine, Aging and Kidney Disease Department, Sant'Orsola-Malpighi University Hospital, Poliambulatorio Pad. 2, Via Albertoni 15, 40138 Bologna, Italy; ^3^Department of Endocrinology, Physiopathology and Applied Biology, Università degli Studi di Milano, 20133 Milan, Italy; ^4^Unit of Endocrinology and Metabolism, Department of Metabolic Diseases, Nutrition and Wellness, INRCA-IRCCS, 00189 Rome, Italy; ^5^Department of Human Morphology and Biomedical Sciences, Università degli Studi di Milano, 20133 Milan, Italy

## Abstract

We evaluated the association of the sex hormone pattern and the serum level of the main adipokines to metabolic syndrome (MS) and its components in 199 pharmacologically untreated subjects. Men and women included in the age-class subgroups were matched for body mass index, waist circumference, blood pressure, heart rate, fasting plasma glucose, and plasma lipids. Men without MS had significantly lower leptin/adiponectin ratio than men with MS. Women without MS had lower leptin and leptin/adiponectin ratio than women with MS but had significantly higher adiponectin, estrone, and dehydroepiandrosterone levels. In men, the leptin/adiponectin ratio is the main factor associated to MS diagnosis (OR: 3.36, 95% CI 1.40–8.08), while in women adiponectin alone appears to be a protective factor (OR: 0.87, 95% CI 0.79–0.95). In conclusion, in a sample of pharmacologically untreated subjects, leptin/adiponectin ratio seems to be the factor more strongly associated to MS and its components.

## 1. Introduction

The metabolic syndrome (MS) is a highly prevalent complex pathophysiological condition associated to increased cardiovascular disease risk. Interestingly, the risk profile related to the metabolic syndrome appears to be different in men and women [1]. This could be at least partly related to the different hormonal pattern and to the different adipose tissue metabolism [2]. In fact, women generally have a larger proportion of body mass as fat and are more likely to accumulate fat subcutaneously and on their lower extremities, whereas men are more likely to accumulate fat in the abdominal region [3]. In addition, women have higher rates of nonesterified fatty acids (NEFAs) reuptake by the adipose tissue. However, they also have greater rates of fat oxidation during prolonged exercise: oestrogens appear to underlie many of these differences [4]. Fat and fertility are linked through leptin in humans, and in women, reduced fertility has been indeed associated with reduced leptin plasma levels [5]. On the other side, leptin secretagogues have similar efficacy in subjects with different body weight, yet significantly higher in women than in men [6]. Moreover, physiological conditions (menopause, and aging) that influence the pattern of sex hormones are also associated to changes in the synthesis and secretion of adipokines, which contribute to the development of MS, target organ damage, and other chronic diseases [7]. Adipokines have actually been related to carotid atherosclerosis [8] and coronary calcification [9]. The interrelationship between sex hormones and adipokine levels has been observed in different studies. Adiponectin was found to be lower in men, in comparison to women, [10] despite an inverse relationship between estradiol and adiponectin, [11] indicating that in addition to estradiol, other gender-dependent factors may be of relevance. The relationship between testosterone and adiponectin is less clear. While in rodents, testosterone injection decreases adiponectin levels, [12] in healthy adult-elderly humans, [13] and in women affected by polycystic ovary syndrome, [14] serum adiponectin and testosterone are directly correlated. However, when compared with eugonadal subjects, hypogonadal men have greater adiponectin levels, which are reduced by testosterone replacement therapy [15]. On the other hand, in males, negative correlations between testosterone and leptin have been clearly shown in different cross-sectional studies, [16, 17] including one from our group [18]. In addition, testosterone therapy reduces serum leptin concentrations in subjects with low testosterone levels [19]. The explanation for such relationship is the presence of functional leptin receptors in reproductive tissues and, reciprocally, of steroid hormone receptors on adipocytes. Although the exact role of leptin is not fully understood, this adipokine is well known to be involved in normal sexual maturation and reproduction [20].

On this basis, the aim of our study was to evaluate the association of the sex hormone pattern and the serum level of the main adipokines to MS and its components in a cohort of pharmacologically untreated subjects.

## 2. Materials and Methods

### 2.1. The Brisighella Heart Study

The Brisighella Heart Study is a prospective, population-based longitudinal epidemiological cohort involving 2939 randomly selected subjects, aged 14 to 84 years, free of cardiovascular disease at enrolment, resident in the Northern Italian rural town of Brisighella. The study was promoted in 1972 by Professor G. Descovich [21]. Subjects were clinically evaluated at baseline and every 4 years following enrolment when extensive clinical and laboratory data were obtained in addition to the assessment of morbidity and mortality. In 1986, the study became part of the WHO European Risk Factors Coordinated Analysis, and in 1990, it became part of the Risk Factors and Life Expectancy Project [22]. Throughout the duration of the entire study, all-cause mortality and morbidity, as well as the incidence of the main cardiovascular risk factors, were recorded. Every three months, the study design included an update of the database with regard to fatal and nonfatal new events and every four years, a complete medical checkup comprised a nutritional habits record and fasting blood sample was performed. From 1986 to 1988, several programs started to check efficacy, cost, and reliability of primary and secondary cardiovascular prevention, including school children and general population nutritional education programs and general practitioner training concerning therapeutic guidelines [21]. Physical activity and nutritional habits have been recorded throughout the study and encoded as previously reported [22]. The study was carried out in agreement with the Declaration of Helsinki. It was approved by the Ethical Committee, and all subjects gave their written consent to be involved in the study.

### 2.2. Subject Selection

For this study, from the database of the historical cohort of the Brisighella Heart Study, we selected adult male and women subjects classified as healthy and free of antihypertensive, lipid-lowering, or antidiabetic drugs, representative of their age, and cross-matched by age, body mass index (BMI), systolic blood pressure (SBP), diastolic blood pressure (DBP), pulse pressure (PP), heart rate (HR), respiratory rate (RR), total cholesterol (TC), low-density lipoprotein cholesterol (LDL-C), triglycerides (TG), and fasting plasma glucose (FPG). HR and RR were included as selection criteria as indirect markers of sympathetic activity that is related to MS and, more generically, overweight [23]. Plasma lipids were included as selection criteria to exclude a priori people affected by metabolic diseases and because we did not select patients on the basis of nutritional habits: since TG (and consequently HDL and LDL) could be strongly influenced by dietary factors, [24] we preferred to standardize patients on the basis of their lipid values as an indirect markers of correct dietary habits.

The final sample included 199 subjects (M:89; F:110), aged 62.5 ± 12.4 years. The main characteristics of the selected subjects are reported in Table 1.

### 2.3. Laboratory Methods

All the assays have been carried out on 12-hour fasting sampled blood. Routine hematochemical analyses have been carried out using standardized methods. Plasma leptin, adiponectin, and testosterone concentrations were measured using enzyme-linked immunoSorbent assay (ELISA) kits from R&D Systems (Minneapolis, Minn, USA) [25]. The lowest limits of sensitivity were 7.8 pg/mL for leptin, 0.246 ng/mL for adiponectin, and 0.030 ng/mL for testosterone. Ghrelin was measured using the human ghrelin (total) ELISA kit from Millipore (St. Charles, Mo, USA) [26]. The lowest level of total ghrelin that can be detected by this assay is 30 pg/mL. Plasma estrone and dehydroepiandrosterone sulphate (DHEAS) levels were detected by ELISA kits from BioVendor (Heidelberg, Germany). The lowest limits of sensitivity were 10.0 pg/mL for estrone and 0.005 *μ*g/mL for DHEAS.

### 2.4. Statistical Analysis

Patients were classified as affected or not by MS on the basis of the NCEP ATPIII guidelines [27]. A full descriptive analysis of the available parameters was carried out, and continuous variables were tested for normality according to the Kolmogorov-Smirnov test. Results were reported as mean ± standard deviation for normally distributed parameters and as median ± 95% confidence intervals for not-normally distributed variables. Not-normally distributed variables were log-transformed in order to carry on advanced statistics. The association of sex hormones, adipokines, and MS and its components has been evaluated by the application of a logistic regression model and the age-adjusted odds ratio (OR) with 95% confidence intervals (CI) have been calculated. The model included all variables not directly related to that considered in the definition of MS. A threshold “*P*” level <0.05 was chosen as significant for all the tests. All analyses were carried out with the help of SPSS 13 version for Windows.

## 3. Results

**Figure 1:**
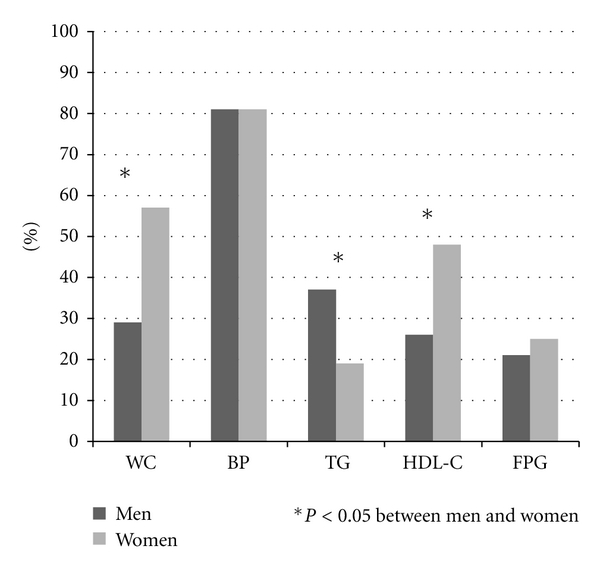
Percentage distribution of metabolic syndrome components between genders.  *P < 0.05 between men and women.

MS was present in 27 (31.4%) men and 45 (43.3%) women (Pearson Chi-Square = 2,82. *P* = 0.093). The percent distribution of the individual MS components has been summarized in Figure 1. Men tended to have TG above the MS diagnostic cutoff whereas women to have waist circumference and HDL-C above and below the diagnostic cutoff, respectively.

Adipokine and sex hormone levels are reported by gender and MS diagnosis in Table 2. Among subjects without MS, men had a leptin/adiponectin ratio significantly lower than women; among subjects with MS, men had significantly lower leptin, adiponectin, and leptin/adiponectin ratio than women, who showed significantly higher estrone, and DHEAS.

Comparing subjects with and without MS within genders, men without MS had significantly lower leptin/adiponectin ratio than men with MS. On the other side, women without MS had lower leptin and leptin/adiponectin ratio than women with MS but had significantly higher adiponectin, estrone and DHEAS levels.

In men, the leptin/adiponectin ratio is the main factor associated to MS diagnosis (OR: 3.36, 95% CI 1.40–8.08), while in women, adiponectin alone appears to be a protective factor against this diagnosis (OR: 0.87, 95% CI 0.79–0.95).

The main factors associated to individual MS criteria are reported in Table 3.

## 4. Discussion

Whereas there are contrasting data about the association of leptin/adiponectin ratio and vascular damage, [8, 28] it is largely accepted that this ratio is strongly associated with insulin resistance, which represents the major feature of the MS [29]. In our study, carried out in a representative sample of pharmacologically untreated adult-elderly subjects involved in the Brisighella Heart Study, we observed that in men, the leptin/adiponectin ratio is the main factor associated to MS diagnosis, while in women, adiponectin alone appears to be a protective factor. Considering the relatively old age of the women participating in our study, this result is in line with what just reported by Milewicz et al. [30].

When the MS components are considered individually, we observed that in men, the leptin/adiponectin ratio acted as the factor more strongly associated to higher blood pressure and triglycerides and lower HDL-C level, while in women, this was best associated to higher blood pressure and waist circumference. Testosterone was strongly associated to high blood pressure in women, while estrone was mildly associated to high fasting plasma glucose in men. 

In fact, testosterone favors visceral fat accumulation, and inflammatory pattern in women, thus possibly enhancing an intrinsic blood pressure increasing trend [31] although some protective effects have been recently postulated by other authors [32]. Should the latter be confirmed by further studies, the correlation between T levels and blood pressure we observed in women might be even interpreted as a compensatory mechanism. 

In men, estrone comes mostly from androgen metabolism within the adipose tissue, and therefore, its parallel increase with fasting plasma glucose might reflect the relative increase in fat accumulation and insulin resistance somehow expected from early steroid physiology studies supported by more recent clinical evidence [33, 34]. Adiponectin seems to be a protective factor against high FPG in women. Interestingly, uric acid, included in the original Reaven's MS definition, [35] appeared to be strongly associated to high waist circumference in men and to the typical atherogenic dyslipidemia in women. 

Overall, these data support the observations showing that a large part of cardiovascular risk factors are related to adipose tissue metabolism but with different features in men and women [36]. These observations are actually relevant when planning preventive interventions in the setting of general population, where they need to be adapted to each gender [37].

Our study has some relevant limitations. The first is the relatively small size of the study cohort. In any case, the subjects have been selected as representative for their age classes in the general population, and they are pharmacologically untreated (in order to avoid interference on the hormonal and adipokine pattern). Moreover, we have limited our research to some sex hormones and adipokines, while we are aware that several other related parameters could be investigated. Besides, at the best of our knowledge, in the present study, we investigated the more widely accepted and cited parameters. Therefore, the lack of differences among groups in ghrelin concentration, previously reported by other authors, [38] could be related to our patients selection, with similar baseline HR and RR: in fact, very recent evidence show that ghrelin modulates the sympathetic activity [39] and our observation is probably an indirect confirmation of these data.

In conclusion, in a sample of pharmacologically untreated adult-elderly subjects, leptin/adiponectin ratio seems to be the factor more strongly associated with MS (especially in men) and its components even if differently in men and women.

## Figures and Tables

**Table 1:** Main clinical and laboratory characteristics of the studied population.

Variable	Men (*N*. 89)		Women (*N*. 110)	
	Mean	SD	Mean	SD
Age (years)	63.55	12.91	61.68	11.88
BMI (kg/m^2^)	27.08	3.37	28.04	4.94
SBP (mmHg)	140.06	18.39	142.82	20.46
DBP (mmHg)	81.84	9.83	83.55	10.70
Pulse pressure (mmHg)	58.22	17.69	59.26	14.40
Heart rate (ppm)	71.12	9.85	72.70	8.84
Respiratory rate (rpm)	20.02	3.18	21.59	6.25
TC (mg/dL)	213.91	30.97	223.80	31.53
TG (mg/dL)	138.45	66.14	128.60	51.20
HDL-C* (mg/dL)	47.43	10.22	52.40	13.84
LDL-C (mg/dL)	138.78	30.64	147.67	31.52
Non-HDL-C (mg/dL)	166.47	32.43	171.39	35.91
FPG (mg/dL)	101.45	25.34	99.19	22.75

*Men versus women, *P* < 0.05.

**Table 2:** Adipokine and sex hormone levels by gender and metabolic syndrome diagnosis.

Variable	No Metabolic syndrome				Metabolic syndrome			
	Men (*N*. 61)		Women (*N*. 28)		Men (*N*. 63)		Women (*N*. 47)	
	Mean	SD	Mean	SD	Mean	SD	Mean	SD
Adiponectin (pg/mL)	10.19	4.78	15.57°	6.86	8.39*	4.90	11.32	5.06
Leptin (pg/mL)	4.67	4.05	13.23°	10.47	6.31*	3.72	19.45	13.34
Leptin/adiponectin	0.54^∗°^	0.48	1.11°	1.14	0.97*	0.76	2.17	1.75
Ghrelin (pg/mL)	758.33	202.98	730.35	230.63	729.32	152.72	724.65	188.84
Testosterone (ng/mL)	7.00	5.28	2.04	1.57	6.46	2.72	1.76	1.32
Estrone (pg/mL)	153.07	139.52	153.21°	142.20	234.79*	250.39	106.32	58.75
DHEAS (*μ*g/mL)	1.10	0.59	0.90°	0.53	1.08*	0.62	0.69	0.43
Testosterone/estrone	0.06	0.04	0.01	0.02	0.05	0.03	0.01	0.01

*P < 0.05 between men and women.

°*P* < 0.05 versus subjects of the same sex without MS.

**Table 3:** Factors associated to the single metabolic syndrome components in men and women.

MS component	Men			Women		
	Variable	OR	95% CI	Variable	OR	95% CI
Waist circumference	Uric acid	2.21	1.3–3.6	L/A ratio	4.43	1.87–10.47
Blood pressure	L/A ratio	4.73	1.01–14.43	Testosterone	2.18	1.09–4.36
				L/A ratio	2.19	1.01–6.02
Fasting plasma glucose	Estrone	1.01	1.01–1.05	Adiponectin	0.86	0.76–0.97
Triglycerides	L/A ratio	2.87	1.23–6.67	Uric acid	1.85	1.18–2.91
HDL-cholesterol	L/A ratio	2.84	1.22–6.61	Uric acid	1.93	1.23–3.01

L/A: Leptin/Adiponectin Ratio.
